# Factors that influence survival in a probable Alzheimer disease cohort

**DOI:** 10.1186/alzrt119

**Published:** 2012-05-15

**Authors:** Susan D Rountree, Wenyaw Chan, Valory N Pavlik, Eveleen J Darby, Rachelle S Doody

**Affiliations:** 1 Alzheimer's Disease and Memory Disorders Center, Department of Neurology, Baylor College of Medicine, 1977 Butler Boulevard, Suite E5.101, Houston, TX 77030, USA; 2 Division of Biostatistics, University of Texas School of Public Health, 1200 Herman Pressler, Suite 846, Houston, TX 77030, USA; 3 Department of Family and Community Medicine, Baylor College of Medicine, 3701 Kirby Drive, Houston, TX 77098, USA

## Abstract

**Introduction:**

This longitudinal study examined multiple factors that influence survival in a cohort of Alzheimer patients followed over two decades.

**Methods:**

Time to death after symptom onset was determined in 641 probable AD patients who were evaluated annually until death or loss to follow-up, and information was entered into a longitudinal database. Date of death was available for everyone including those eventually lost. Baseline variables included age, sex, race, disease severity, a calculated index of rate of initial cognitive decline from symptom onset to cohort entry (pre-progression rate or PPR), years of education, and medical comorbidities (diabetes, hypertension, hyperlipidemia, coronary disease, cerebrovascular disease). Multivariable Cox proportional hazard regression analysis was used to analyze the baseline and/or time dependent association in Mini-mental Status Exam (MMSE) severity, Physical Self Maintenance Scale (PSMS), Persistency Index (PI) of exposure to antipsychotic and antidementia drugs, and psychotic symptoms (hallucinations, delusions) with mortality.

**Results:**

Baseline covariates significantly associated with increased survival were younger age (p = .0016), female sex (p = .0001), and a slower PPR (p < .0001). Overall disease severity at baseline, medical comorbidities, and education did not influence time to death. Time-dependent changes in antipsychotic drug use, development of psychotic symptoms, antidementia drug use, and observed MMSE change were not predictive. In the final model the only time-dependent covariate that significantly decreased survival was worsening of functional ability on the PSMS (hazard ratio = 1.10; CI: 1.07-1.11).

**Conclusions:**

In this large AD cohort survival is influenced by age, sex, and the development of functional disability during follow-up. The most important predictor of mortality was a faster rate of cognitive decline at the initial patient visit (PPR). The currently available antidementia drugs do not prolong survival in Alzheimer patients.

## Introduction

Life expectancy in people with Alzheimer's disease (AD) is, overall, shorter than what is expected in age-matched, cognitively normal seniors and may be influenced by age, disease severity, general debility, extrapyramidal signs, gender, and race or ethnicity [1-4]. Antipsychotic drugs have also been linked to increased risk of death in older people with dementia [5-7]. Estimating survival following the recognition of AD is an important health matter for patients and their families who must plan for medical care at the end of life. Estimating length of life is also important for predicting the impact of dementia on health-care resources [8].

Since 1993, five drugs have been marketed in the US for the treatment of AD (donepezil, galantamine, rivastigmine, tacrine, and memantine). Antidementia drugs have been proven to mitigate the symptoms of AD but their influence on long-term course and life span is not established. Recent observational studies suggest that cognitive and functional benefits continue over many years for patients who persist in their treatment, beyond the relatively short duration of benefit evident in clinical research trials [9-11]. Antidementia drugs are not thought to influence longevity, but there are conflicting reports in the literature. Several observational studies [11-14] found no relationship between the use of any antidementia drug regimen (cholinesterase inhibitor or memantine or both) and survival when users were compared with untreated patients. Two large cross-sectional studies that involved retrospective data analysis reported that the use of cholinesterase inhibitors versus no treatment significantly increased survival in nursing home patients. Both tacrine use [15] (hazard ratio = 0.76, confidence interval (CI) = 0.70 to 0.83) and donepezil use [16] (hazard ratio = 0.89, 95% CI = 0.83 to 0.95) were associated with significantly reduced mortality. This study evaluated a broad range of covariates suspected to influence survival and assessed the use of antidementia drugs in a time-dependent analysis.

## Materials and methods

### Participants

Informed consent was received from all patients involved in the study. The patients were evaluated at the Baylor College of Medicine Alzheimer's Disease and Memory Disorders Center. The study began in 1989 and enrolled 1,833 patients with dementia as of 31 December 2005 (censoring date). All members of this community-based cohort agreed to participate in a database approved by the institutional review board of the Baylor College of Medicine. Six hundred forty-one participants met the established criteria for probable AD as determined by the National Institute of Neurological and Communicative Disorders and Stroke and the Alzheimer's Disease and Related Disorders Association (now known as the Alzheimer's Association) [17] and were included in this analysis. Vital status is obtained from the National Death Index every 6 months and allows calculation of survival time for all enrolled subjects.

### Exposure

Cumulative drug exposure to antidementia drugs (cholinesterase inhibitors or memantine or both) or antipsychotic drugs (typical or atypical) was determined from the onset of symptoms. The onset of first symptoms is estimated by a physician using a standardized algorithm to the nearest half-year [18]. Start and stop dates of drug exposure are recorded at the first clinic visit by history obtained from the patient and caregiver along with a review of medical and pharmacy records. This information is updated at each return visit to the center. All periods on a given drug are summed in order to generate the cumulative drug exposure. Lapses in treatment or switching from one drug to another are recorded. To reconcile antidementia drug exposure that occurred by virtue of participation in a clinical research trial, we obtained the blinding data from those trials. No attempt was made to quantify the dose of medication or distinguish between drug regimens (for example, monotherapy or combination therapy with a cholinesterase inhibitor and memantine or use of any particular antipsychotic drug).

### Covariates

Baseline variables were age, sex, race (white versus non-white), disease severity based upon Mini-Mental Status Examination (MMSE) score [19], years of formal education, medical comorbidites present in the past or currently active (diabetes, hypertension, hyperlipidemia, coronary disease, or cerebrovascular disease), and the pre-progression rate (PPR) [20], a calculated rate of cognitive decline prior to enrollment. Patients with AD progress at intrinsically different rates, but little is known about factors that explain the variance. The PPR has prognostic value in classifying patients as rapid, intermediate, or slow progressors [20]. It is calculated at the initial clinic visit by means of the following formula: (the MMSE score out of 30 - initial MMSE score) / physician's estimate of symptom duration (in years). Patients are stratified into slow (decline of 0 to 1.9 MMSE points per year), intermediate (decline of 2 to 4.9 MMSE points per year), or rapid (decline of at least 5 MMSE points per year) progressors.

We used a time-dependent mechanism to assess the impact of changes in cognition measured by the MMSE, basic activities of daily living measured by the Physical Self-Maintenance Scale (PSMS) [21], time-dependent changes in the Persistency Index (PI) or exposure to antipsychotic and antidementia drugs, and the development of psychotic symptoms (hallucinations and delusions) on time to death. The PI is calculated as the total duration of drug treatment (in years) divided by the total duration of symptoms (in years) extended to the censoring date or death [10]. Only a few participants developed medical comorbidities following baseline evaluation and so it was not possible to use a time-dependent mechanism to assess this variable.

### Statistical analysis

Time to death for all-cause mortality was evaluated by multivariable Cox proportional hazard regression analysis with stepwise selection process to evaluate baseline and time-dependent change in covariates or risk factors. All analyses were performed by using SAS version 9.2 (SAS Institute Inc., Cary, NC, USA). Logistic regression was applied to determine the covariates significantly associated with survival (determined by a *P *value of not more than 0.05), and hazard ratios for significant covariates were determined in the final model.

## Results

Median survival time among the 641 patients with probable AD following the onset of symptoms was 11.3 years (CI = 10.4 to 11.8), and there were 352 deaths. The mean (standard deviation) follow-up time after the baseline visit to censoring or death was 3.0 (1.94) years; overall, the patients in this cohort returned to the clinic for an average of 2.4 (1.64) visits. The cohort predominately was female (68%) and white (87%). Many participants were already using antidementia drugs prior to the initial visit (42.6%). The average baseline MMSE score was 19.5 (6.64), and the range was 0 to 30; the average baseline PSMS score was 7.9 (3.05), and the range was 6 to 25 (Table 1).

**Table 1:** Population characteristics of patients with Alzheimer's disease

Variable (n = 641)	Value	Percentage	Range
Age, years	73.0 (8.50)		43-93
Female		68	
Race (white versus non-white)		87	
Education, years	14.0 (3.56)		0-29
Duration of symptoms before initial visit, years	3.7 (2.29)		0.5-13
Follow-up time from baseline to censoring or death, years	3.0 (1.94)		0.7-13.4
Total number of follow-up visits to the clinic	2.4 (1.64)		2-11
MMSE score	19.5 (6.64)		0-30
PSMS score	7.9 (3.05)		6-25
Using antipsychotics drugs	221	34.5	
Using antidementia drugs	554	86.4	
Pre-progression rate			
Slow	192	30.0	
Moderate	297	46.3	
Fast	152	23.7	
Experiencing hallucination	291	45.4	
Experiencing delusions	371	57.9	

Continuous variables are presented as mean (standard deviation). MMSE, Mini-Mental Status Examination; PSMS, Physical Self-Maintenance Scale.

The assumption of proportionality is met when age, gender, severity, and baseline MMSE are included. Increasing age (hazard ratio = 1.03 per year, 95% CI = 1.01 to 1.04), male gender (hazard ratio = 1.72, 95% CI = 1.31 to 2.26), and faster rate of cognitive decline at baseline as measured by the PPR category - hazard ratios were 0.45 (slow versus rapid), 0.75 (slow versus intermediate), and 0.59 (intermediate versus rapid), and 95% CIs were 0.30 to 0.66, 0.54 to 1.04, and 0.43 to 0.82, respectively - were significantly associated with increased risk of death (Table 2). Severity of AD and medical comorbidities were not associated with survival in the univariate analysis or in the age- and gender-adjusted analysis. In the final model, race (white versus non-white), presence or history of medical comorbidities, baseline disease severity (mild or moderate versus severe stage disease), and years of formal education did not influence survival. The development of functional impairment in basic activities of daily living as measured by the PSMS was associated with significantly increased risk of death (hazard ratio = 1.10, CI = 1.08 to 1.11) (Table 2). Time-dependent change in the use of either antidementia drugs or antipsychotic drugs, progression of disease severity measured by the MMSE, and the development of psychosis (hallucinations or delusions) did not influence survival in the final model.

**Table 2:** Factors associated with increased risk of death

Covariates	*P *value	Hazard ratio	95% CI
Age	0.0017	1.03	1.01-1.04
Male	< 0.0001	1.72	1.31-2.26
Pre-progression category	0.0002		
Slow versus intermediate		0.75	0.54-1.04
Slow versus rapid		0.45	0.30-0.66
Intermediate versus rapid		0.59	0.43-0.82
Time-dependent change in ADLs or worsening PSMS score	< 0.0001	1.10	1.07-1.11

ADLs, activities of daily living; CI, confidence interval; PSMS, Physical Self-Maintenance Scale.

## Discussion

The median survival time of this cohort with probable AD diagnosis was 11.3 years from the onset of symptoms. This figure may overestimate the length of survival in AD since individuals with rapidly progressive illness may die before they obtain a diagnosis. Median survival time in a Canadian study that evaluated survival from the onset of symptoms of dementia found that patients with AD had a 3.1-year median survival time after correction for the so-called length bias [22], but the population was much older than ours; the average age was 83.8 (7.03) years. Survival from onset of symptoms was not modified by white or non-white race or education. All non-white races/ethnicities were reported to have a survival advantage following diagnosis over white patients in a large retrospective analysis [3], but the present study may have been underpowered to detect small differences in survival between these two groups. Survival in AD in a study of incident cases also found no differences in mortality by race or ethnicity but did report that a history of diabetes or hypertension was associated with a shorter life span [23], whereas our study did not confirm the risk of death due to these comorbidities (as discussed below). Our results were in agreement with those of another study [24] that evaluated cognitive decline and survival in patients with AD and found no relationship between survival and educational attainment.

Our results confirm previous findings that some of the factors that predict survival in the general population are also relevant to AD; specifically, several of the main predictors for survival in AD are age [8] and sex [1,4,25,26] along with an impairment or decline in functional abilities [27,28]. Every 1-point increase on the PSMS, which measures the ability to perform basic activities of daily living and is scored on a scale of 0 to 30 points, was associated with an increased risk of death of 10% per year.

Like the investigators in a large population study that was performed in the UK and that used multivariable adjustment [29], we found that disease severity is not associated with survival. Neither disease severity at baseline nor time-dependent changes in the MMSE score influenced survival. However, the PPR indicates the rate at which a patient declines following the onset of symptoms, presumably an intrinsic disease progression rate, and was significantly associated with increased risk of death. We previously reported that patients who are slow progressors have significantly reduced mortality compared with fast progressors (hazard ratio = 0.62, 95% CI = 0.43 to 0.91, *P *= 0.024), but the mortality between intermediate and fast progressors did not reach significance in that study (hazard ratio = 0.81, 95% CI = 0.59 to 1.15, *P *= 0.24) [27]. The present study suggests that the survival advantage associated with the PPR is on a continuum and not limited to those with slowest disease progression.

It is often presumed that medical comorbidities should also influence survival with AD. Diabetes, hypertension, hyperlipidemia, coronary disease, and cerebrovascular disease at baseline did not influence survival in this cohort. This result was similar to that of another study [28] and could be a consequence of length bias or the fact that patients with severe medical comorbidity may never seek treatment for dementia. The study which did find that these comorbidites reduced survival [25] did not examine the other covariates used in our analysis.

Although studies suggest that atypical antipsychotic drugs increase risk of death in older patients with dementia [7] and the use of traditional or typical agents is associated with even greater risk of death [5,6], we could not replicate these findings in our outpatient-based sample. Time-dependent changes in the use of antipsychotic drugs or psychotic symptoms (hallucinations or delusions) did not impact survival in this analysis. Our results are consistent with those of a cross-sectional study with longitudinal follow-up, in which neither the use of antipsychotic medications nor the development of psychosis increased risk of death [30]. Psychosis was reported to be associated with a more rapid disease progression or functional decline in another study but again did not increase risk of death [31]. Our results call into question the suggestion that antipsychotic drugs prescribed to patients with dementia will shorten their life span, but an important difference may be that most of our patients lived in the community rather than in nursing homes. Additionally, our patients are treated with low doses of antipsychotic drugs, which may not confer the same risk as higher doses included in prior studies.

The majority of caregivers for patients with AD identify quality of life and preservation of patient cognition and function as being the most important benefits to be derived from therapy [32]. Previous pivotal drug studies have demonstrated drug-placebo benefits, and observational studies support the long duration of these benefits [9-11,33]. Our findings support the view that patients with mild, moderate, or severe AD can be treated without the worry that such treatment will prolong life in the most debilitated stages.

## Conclusions

In this large AD cohort, survival is influenced by age, sex, and a calculable intrinsic rate of decline. Disease severity at baseline, vascular risk factors, and years of education did not influence time to death. Time-dependent changes in antipsychotic drug use or development of psychotic symptoms, antidementia drug use, and observed MMSE score change were not predictive. The only time-dependent covariate that significantly decreased survival was worsening of functional abilities. Currently available antidementia drugs provide cognitive and functional benefit yet do not prolong overall survival in patients with AD.

## Abbreviations

AD: Alzheimer's disease; CI: confidence interval; MMSE: Mini-Mental Status Examination; PI: Persistency Index; PPR: pre-progression rate; PSMS: Physical Self-Maintenance Scale.

## Competing interests

In the past five years, SDR has received investigator-initiated grant funding from the Forest Laboratories, Inc. (New York, NY, USA) and honoraria from the Forest Laboratories, Inc, Pfizer Inc (New York, NY, USA), and Novartis (Basel, Switzerland) for speaking at non-CME (non-continuing medical education) events. In the past five years, RSD has received honoraria for serving as a consultant to Novartis and Pfizer Inc in regard to general drug development and honorarium from the Forest Laboratories, Inc for attending an advisory meeting; her institution has received payments from Janssen Alzheimer Immunotherapy (Dublin, Ireland) and Pfizer Inc for performing clinical trials on unmarketed drugs, for which she is the principal investigator. The other authors declare that they have no competing interests.

## Authors' contributions

SDR participated in research design and analysis of data and wrote the manuscript. WC and EJD performed data analysis. VNP participated in revision of the manuscript. RSD participated in obtaining funding, analysis of data, and revision of the manuscript. All authors read and approved the final manuscript.
